# Membrane Transfer from Mononuclear Cells to Polymorphonuclear Neutrophils Transduces Cell Survival and Activation Signals in the Recipient Cells via Anti-Extrinsic Apoptotic and MAP Kinase Signaling Pathways

**DOI:** 10.1371/journal.pone.0156262

**Published:** 2016-06-03

**Authors:** Ko-Jen Li, Cheng-Han Wu, Chieh-Yu Shen, Yu-Min Kuo, Chia-Li Yu, Song-Chou Hsieh

**Affiliations:** 1 Institute of Clinical Medicine, National Yang-Ming University College of Medicine, Taipei, Taiwan; 2 Institute of Clinical Medicine, National Taiwan University College of Medicine, Taipei, Taiwan; 3 Institute of Molecular Medicine, National Taiwan University College of Medicine, Taipei, Taiwan; 4 Department of Internal Medicine, National Taiwan University Hospital, Taipei, Taiwan; INSERM-Université Paris-Sud, FRANCE

## Abstract

The biological significance of membrane transfer (trogocytosis) between polymorphonuclear neutrophils (PMNs) and mononuclear cells (MNCs) remains unclear. We investigated the biological/immunological effects and molecular basis of trogocytosis among various immune cells in healthy individuals and patients with active systemic lupus erythematosus (SLE). By flow cytometry, we determined that molecules in the immunological synapse, including HLA class-I and-II, CD11b and LFA-1, along with CXCR1, are exchanged among autologous PMNs, CD4^+^ T cells, and U937 cells (monocytes) after cell-cell contact. Small interfering RNA knockdown of the integrin adhesion molecule CD11a in U937 unexpectedly enhanced the level of total membrane transfer from U937 to PMN cells. Functionally, phagocytosis and IL-8 production by PMNs were enhanced after co-culture with T cells. Total membrane transfer from CD4^+^ T to PMNs delayed PMN apoptosis by suppressing the extrinsic apoptotic molecules, *BAX*, *MYC* and caspase 8. This enhancement of activities of PMNs by T cells was found to be mediated via p38- and P44/42-Akt-MAP kinase pathways and inhibited by the actin-polymerization inhibitor, latrunculin B, the clathrin inhibitor, Pitstop-2, and human immunoglobulin G, but not by the caveolin inhibitor, methyl-β-cyclodextrin. In addition, membrane transfer from PMNs enhanced IL-2 production by recipient anti-CD3/anti-CD28 activated MNCs, and this was suppressed by inhibitors of mitogen-activated protein kinase (PD98059) and protein kinase C (Rottlerin). Of clinical significance, decreased total membrane transfer from PMNs to MNCs in patients with active SLE suppressed mononuclear IL-2 production. In conclusion, membrane transfer from MNCs to PMNs, mainly at the immunological synapse, transduces survival and activation signals to enhance PMN functions and is dependent on actin polymerization, clathrin activation, and Fcγ receptors, while membrane transfer from PMNs to MNCs depends on MAP kinase and PKC signaling. Defective membrane transfer from PMNs to MNCs in patients with active systemic lupus erythematous suppressed activated mononuclear IL-2 production.

## Introduction

Polymorphonuclear neutrophils (PMNs) defend against bacterial invasion and interact via cytokines with other immune cells, including lymphocytes, antigen-presenting cells (APC), monocytes/macrophages and natural killer (NK) cells [1–4]. In PMN-depleted rats, delayed-type hypersensitivity and tumor inhibitory functions are suppressed, whereas humoral immune responses are enhanced [5–8]. Interestingly, interferon (IFN)-γ, interleukin (IL)-3 and granulocyte-macrophage colony-stimulating factor can induce PMN to express major histocompatibility complex (MHC) class-II and the T cell co-stimulatory molecules CD80 and CD86, enabling them to act as APC, and enhance T cell proliferation [9–11]. Furthermore, PMNs may trans-differentiate into dendritic-like cells at sites of chronic rheumatoid synovitis and granulomatosis with polyangiitis [12, 13]. Thus, PMNs modulate diverse immune functions of mononuclear cells (MNCs). However, the molecular basis of PMN-MNC interactions, other than those involving cytokines, remains unclear.

Intercellular membrane transfer, or trogocytosis, via immunological synapses is important in cell-cell communication [14–19]. During cell-cell contact, CD4^+^ T cells recognize molecules expressed on APC, including MHC-peptide complexes, CD80 or OX40L [14,15]. The capture of target cell membrane fragments by NK cells is mediated by Src kinase, ATP, Ca^2+^, PKC and a rearranged actin cytoskeleton [16]. Moreover, membrane transfer that occurs spontaneously, without antigen stimulation, among certain homotypical leukemia cell lines has been shown to prolong cell survival [17]. It is conceivable that antibody-dependent PMN-mediated cytotoxicity may play an important role in the control of malignant diseases. Horner et al. [20] demonstrated that trogocytosis during contact between PMNs and target cells can be enhanced in the presence of tumor target antibodies resembling trogocytosis. Our previous research demonstrated that PMN in peritoneal exudate from autoimmune MRL-lpr/lpr mice exerted abnormal effects on Th1/Th2 cytokine profiles, unlike those of normal BALB/c mice [21]. Furthermore, surface-expressed lactoferrins on PMNs are transferred to CD4^+^ T cells, leading to alteration of their cytokine production [22]. We also noted that reduced lactoferrin expression on PMN of patients with active systemic lupus erythematosus (SLE) abnormally modulates Th1/Th2 cytokine production by autologous CD4^+^ T cells [22]. De Toro et al. [23] demonstrated that PMNs can modulate other immune cell functions via the release of cytokines/chemokines [2] or exosomes [23]. These data indicate that PMNs work as important afferent, as well as efferent, cell components in the immune network. In this study, we investigated the proportions of normal PMNs, CD4^+^ T cells, and monocytes/macrophages engaged in trogocytosis, the functional alterations of cells after trogocytosis, and the molecular basis of these. In addition, the comparative membrane transfer from PMNs to MNCs and IL-2 production by recipient cells in patients with active SLE were also explored.

## Materials and Methods

### Study subjects

Healthy volunteers and patients with active SLE, fulfilling SLEDAI-2000 criteria [24], with disease activity scores ≥ 8 were recruited according to a protocol approved by the Institution Review Board and Ethical Committee of National Taiwan University Hospital, Taipei, Taiwan. Each participant provided written informed consent.

### Cell preparation from human peripheral blood

Heparinized venous blood was mixed with one-fourth volume of 2% dextran solution (MW 464,000 Da) (Sigma-Aldrich, St. Louis, MO, USA) and incubated at room temperature for 30 min to sediment red blood cells (RBCs), which served as negative control cells. Leukocyte-enriched supernatant was collected and layered on Ficoll-Hypaque density gradient solution (specific gravity 1.077–1.078; Pharmacia Biotech, Uppsala, Sweden), followed by centrifugation at 250 x *g* for 25 min. MNCs were aspirated from the interface and PMNs retrieved from the pellet. RBCs in the PMN suspension were lysed using chilled 0.83% ammonium chloride solution at 4°C for 10 min. PMNs were then positively selected by monoclonal anti-Gr-1 antibody-conjugated microbeads (derived from the RB6.8C5 hybridoma) and an AutoMACS Pro Separator (Miltenyi Biotec, Bergisch Gladbach, Germany). The MNC suspension was positively selected for monocytes using anti-CD14-conjugated microbeads and an AutoMACS Pro Separator. Remaining lymphocytes were further positively selected using anti-CD4-conjugated microbeads and an AutoMACS Pro Separator. The cell concentrations of RBCs, PMNs, monocytes and CD4^+^ T lymphocytes were adjusted to 2 x 10^6^/ml in in RPMI-1640 containing 10% fetal bovine serum, unless specifically stated otherwise. Trypan blue dye exclusion and FACSort flow cytometry (Becton Dickinson Immunochemistry Systems, Mountain View, CA, USA) were used to confirm > 95% viability and purity of PMNs, monocytes and CD4^+^ T lymphocytes.

### Confocal laser scan microscopic and flow cytometric analysis of membrane exchange

PMNs, CD4^+^ T cells, monocytes or U937 cells (a human macrophage cell line) were surface-stained with either PKH-67 (green fluorescence) or PKH-26 (red fluorescence) (Sigma-Aldrich). After several washes, two of the three cell types were mixed 1:1, centrifuged at 240 x *g* for 1 min to facilitate cell-cell contact or cultured separately in transwell chambers, and incubated at 37°C in 5% CO_2_, 95% air for 1–2 h. For quantification, the percentage and mean fluorescence intensity (MFI) of total non-specific membrane transfer by trogocytosis between two cell types were determined by flow cytometry (Becton Dickinson). To detect specific exchanged molecules, 1:100 dilutions of antibodies against human leukocyte antigen (HLA) class-I, class-II, CD11b, LFA-1, or CXCR1 (all from Santa Cruz Biotechnology, CA, USA), were pre-reacted with one cell type, before co-culture with a second cell type for 1 h. FITC-conjugated goat anti-mouse IgG (diluted 1:2000) was used as a secondary antibody. For confocal laser scan microscopic observation, cells were gently shaken, fixed, and plated on glass slides after incubation. In some experiments, lineage-specific antibodies, including FITC-anti-CD4 for CD4^+^ T cells and FITC-anti-CD16 for PMNs, with DAPI nuclear staining, were applied to co-cultured PKH-26-labeled PMNs or CD4^+^ T cells for 1 h. Trogocytosis between cell types was observed by confocal microscopy.

### Knockdown of the adhesion molecule CD11a in U937 using siRNA

Because expression of CD11b molecules on the surface of U937 cells is modest, we investigated the effects of CD11a on total trogocytosis using small interfering RNA (siRNA; Dharmacon, Lafayette, CO, USA) to knockdown CD11a mRNA expression. CD11a siRNA was transfected by electroporation and CD11a expression in U937 cells quantified by immunoblotting and densitometry. Total non-specific membrane transfer from U937 cells to PMNs was quantified by flow cytometry, as described above.

### Detection of PMN phagocytosis and IL-8 production after cell-cell contact with autologous CD4^+^ T cells

Freshly isolated PMNs were mixed 1:1 with autologous CD4^+^ T cells, followed by centrifugation at 240 x *g* for 1 min. PMN phagocytosis was detected by flow cytometry at 488 nm excitation after 1–2 h incubation with fluorescent beads, as previously described [25–27]. IL-8 concentration in supernatants co-cultured for 24 h was measured using human ELISA kits (Abcam, USA) with a minimal detectable level of 2 pg/ml.

### Application of membrane inhibitors and determination of their effects on PMN function

Membrane inhibitory agents, including the actin polymerization inhibitor latrunculin B (1 μM), the caveolin inhibitor methyl-β-cyclodextrin (MβCD, 5 μM), the clathrin inhibitor Pitstop-2 (30 μM) and the IgGFc receptor blocker human IgG (5 mg/ml), were pre-incubated with PMNs at 37°C for 60 min before mixing with MNCs. The effects on total membrane transfer (measured as MFI), phagocytosis (%), and IL-8 production (pg/ml) were detected as described above.

### Detection of PMN apoptosis after cell-cell contact with autologous MNC

Cell death and early apoptosis of PMNs after cell-cell contact or transwell co-culture with autologous monocytes, lymphocytes, or erythrocytes for 24 h were assessed by double staining with propidium iodide and FITC-annexin V. After washing, cells were analyzed by FACSort flow cytometry, as described above.

### Fluorochrome-labeled inhibitor of caspase probes (FLICA) detection of cytoplasmic caspase 8 and caspase 9 expression in PMNs after co-culture with MNCs

To determine whether the inhibitory effects of PMN-MNC interactions on apoptosis operate via the intrinsic caspase 9 or extrinsic caspase 8 pathways, flow cytometry was used to quantify FLICA- labeled caspase 8 and caspase 9. Freshly-isolated PMNs and MNCs were co-cultured in cell-cell contact or transwell chambers for 24 h. After washing, fresh FLICA reagent (FAM-LEHD-FMK, FLICA CaspaTag, Chemicon, Merck Millipore, Darmstadt, Germany) was incubated with PMNs at 37°C in 5% CO_2_/95% air for 1 h. PMN fluorescence was detected by flow cytometry.

### RT-PCR for mRNA expression of pro-apoptotic and anti-apoptotic genes in PMN after co-culture with CD4^+^ T cells

#### Total cellular RNA extraction and cDNA synthesis

Total cellular RNA was extracted from PMNs (1 x 10^7^ cells/ml) after 2 h co-culture with the same concentration of CD4^+^ T cells or monocytes in conical tubes or transwell chambers, using the Ultraspect RNA isolation kit (Biotex Lab, Houston, TX, USA). cDNA was synthesized using standard methods.

#### PCR amplification of cDNA

An aliquot of cDNA was amplified using oligonucleotide PCR primer pairs specific for human *BCL2L1* (BCL-XL), *BCL2*, *BAX* and *MYC* (c-MYC), in a HYBAID OmniGene DNA Thermal Cycler (HYBAID, Teddington, UK). Human *G3PDH* mRNA expression was used as an internal control. Table 1 shows the nucleotide sequences of primer pairs (Invitrogen, Carlsbad, CA, USA). Amplified cDNA fragments were 127 bp for *BCL2*, 129 bp for *BCL2L1*, 479 bp for *MYC*, 487 bp for *BAX*, and 452 bp for *G3PDH*. These PCR products were electrophoresed in 1.8% agarose gels, alongside ΦX174 digested with the *Hae*III enzyme as a size calibration marker.

**Table 1:** Nucleotide sequences of primer pairs used in this study.

Human mRNA		Primer sequence
*BCL2*	sense	5’-GAG ACA GCC AGG AGA AAT CA-3’
	anti-sense	5’-CCT GTG GAT GAC TGA GTA CC-3’
*BCL2L1*	sense	5’-GGA TGG CCA CTT ACC TGA-3’
	anti-sense	5’-CGG TTG AAG CGT TCC TG-3’
*MYC*	sense	5’-TAC CCT CTC AAC GAC AGC AGC TCG CCC AAC TCC T-3’
	anti-sense	5’-TCT TGA CAT TCT CCT CGG TGT CCG AGG ACC T-3’
*BAX*	sense	5’-CAT CTT CTT CCA GAT GGT GA -3’
	anti-sense	5’-GTT TCA TCC AGG ATC GAG CAG -3’
*G3PDH*	sense	5’-ACC ACA GTC CAT GCC ATC AC-3’
	anti-sense	5’-TCC ACC ACC GG TTG CTG TA-3’

### Western blot detection of PMN intracellular signaling molecules after trogocytosis with lymphocytes or monocytes

Freshly isolated PMNs were mixed 1:1 with either lymphocytes or monocytes in conical tubes, or co-cultured in transwell chambers for 2 h or 14 h. PMNs were then isolated by Ficoll-Hypaque density gradient centrifugation. Total PMN cell lysates were separated by 10% SDS-PAGE and probed using monoclonal antibodies against p38, phospho-p38 MAPK, p44/42, phospho-p44/42 MAPK, Akt, phospho-Akt, IκB, or phospho-IκBα (all from Cell Signaling, Danvers, MA, USA); then, they were reacted with horseradish peroxidase-conjugated anti-mouse IgG as the secondary antibody. Signals were visualized by enhanced chemiluminescence protein detection (Amersham International).

### Evaluation of the effects of various signaling pathway inhibitors on total membrane transfer from PMNs to MNCs

Freshly isolated MNCs were mixed with autologous PKH-67 stained PMNs in a conical tube at 37°C in 5% CO_2_/95% air for 30 min in the presence of different concentrations of various specific signaling pathway inhibitors, including Rottlerin (100 μM), PP2 (10 μM), NLA (100 μM), EGTA (100 μM), Thapsigargin (100 nM), Wortmannin (100 nM), and PD98059 (100 μM) (all obtained from Sigma-Aldrich). Total membrane transfer from PMNs to MNCs was measured by flow cytometry.

### ELISA measurement of IL-2 production by activated MNCs after PMN-MNC co-culture

For co-cultures, autologous PMNs and MNCs obtained from healthy volunteers or patients with active SLE were stimulated with anti-CD3 and anti-CD28 antibodies. After 24 h, supernatants were collected and assayed for IL-2 by ELISA following the manufacturer’s protocol.

### Statistical analyses

Results are presented as mean ± standard deviation. Continuous variables were analyzed by non-parametric Wilcoxon rank-sum test using SPSS for Windows, Version 16.0. Chicago, SPSS Inc. *P*-values < 0.05 were considered statistically significant.

## Results

### Spontaneous membrane exchanges between PMN and autologous CD4^+^ T cells after cell-cell contact

**Fig 1:**
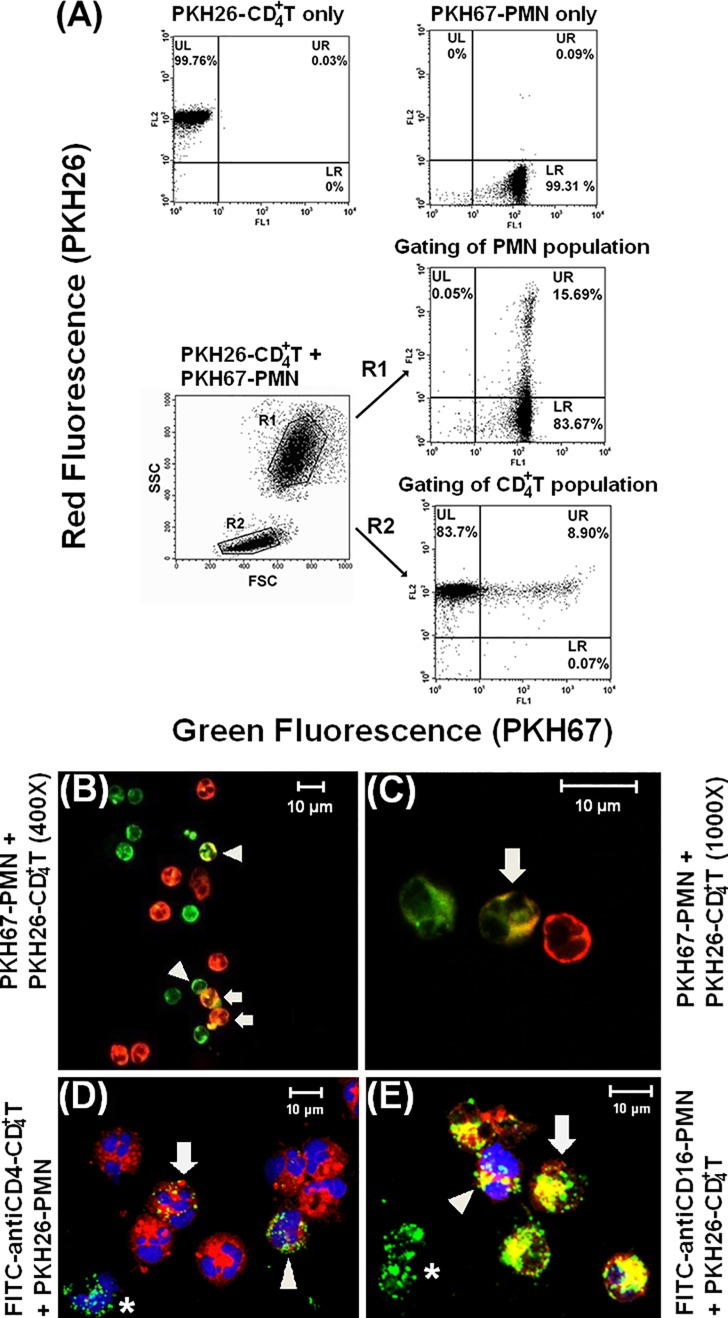
Detection of Trogocytosis. Flow cytometric (A) and confocal laser scan microscopic (B–E) detection of trogocytosis between PKH-26 (red fluorescence)-labeled CD4^+^ T cells and PKH-67 (green fluorescence)-labeled PMN. (A) The proportion of trogocytosis in PMN (panel R1; double stain in UR) and CD4^+^ T (panel R2) detected by flow cytometry after PMN-CD4^+^ T co-culture. (B) Trogocytosis between PMNs (arrows) and CD4^+^ T cells (arrowheads) leads to the generation of yellow fluorescence (magnification x400). (C) Membrane transfer from PKH-26-labeled CD4^+^ T cells to PKH-67-labeled PMNs (arrow) leads to the generation of yellow fluorescence at the site of cell-cell close contact (magnification x1000). (D) Trogocytosis between FITC-anti-CD4 antibody stained CD4^+^ T cell (arrowhead) and PKH-26-labeled PMN (arrow) demonstrating yellow fluorescence. *Indicates FITC-anti-CD4 antibody stained CD4^+^ T cells with no evidence of trogocytosis. Both cell types were nuclear stained with DAPI (blue fluorescence). (E) Trogocytosis between FITC-anti-CD16 antibody-stained PMN (arrow) and DAPI/PKH-26-labeled CD4^+^ T cell (arrowhead) demonstrating yellow fluorescence. *Indicates FITC-anti-CD16 antibody stained PMNs with no evidence of trogocytosis. Images are representative from three independent experiments.

Trogocytosis between PKH-67 (green fluorescence)-labeled PMNs and PKH-26 (red fluorescence) -labeled CD4^+^ T cells was initially detected by flow cytometry (Fig 1A). Approximately 15.69% of the PMN population (gated in R1) captured cell membrane from CD4^+^ T cells. By contrast, only 8.9% of the CD4^+^ T cell population (gated in R2) captured cell membrane from PMN. Spontaneous trogocytosis between autologous PKH-67-labeled PMNs (indicated by arrows in Fig 1B–1E) and PKH-26-labeled CD4^+^ T cells (indicated by arrowheads) was further confirmed by confocal microscopy at 400X (Fig 1B) and 1000X (Fig 1C) magnification. Notably, membrane transfer occurred at the site of intimate contact between PMN and CD4^+^ T cells. Furthermore, lineage-specific CD4 molecules from T cells and CD16 molecules from PMN were also transferred when the two cell types were mixed for 1 h (Fig 1D and 1E). Fig 1D shows that a PKH-26-labeled PMN (arrow) has captured FITC-CD4 molecules from CD4^+^ T cells, while FITC-anti-CD4^+^ T cells (arrowhead) have captured red fluorescence from PMN. Conversely, in Fig 1E PKH-26-labeled CD4^+^ T cells (arrowhead) have captured FITC-labeled CD16 molecules from PMN, and a FITC-anti-CD16 stained PMN (arrow) has captured red fluorescence from PKH-26-labeled CD4^+^ T cells. In summary, bi-directional trogocytosis occurs between autologous PMNs and CD4^+^ T cells after cell-cell contact.

### Trogocytosis between PMN and the macrophage cell line U937 results in the transfer of HLA-II from U937 cells to PMNs and is dependent on cell-cell contact but independent of the adhesion molecule CD11a

**Fig 2:**
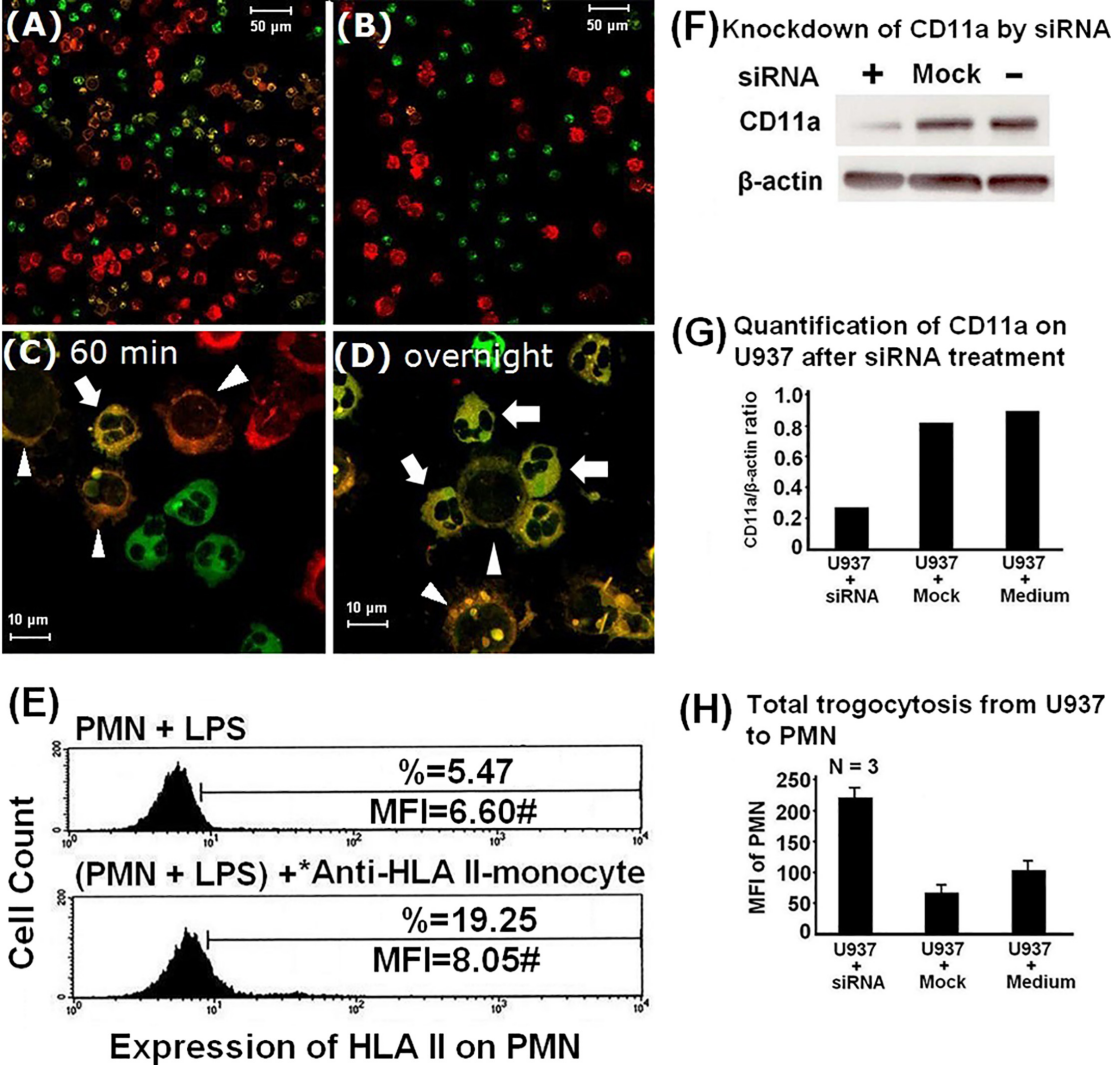
Trogocytosis between PMN and U937 macrophage cells with or without siRNA knockdown of CD11a. Trogocytosis between PKH-67 (green fluorescence)-labeled PMN and PKH-26 (red fluorescence)-labeled-U937 macrophage cells observed by confocal laser scan microscopy. (A) Direct cell-cell contact of PMNs with U937 cells for 1 h, demonstrating yellow fluorescence in many cells (magnification x400) (B) Transwell co-culture of PKH-67-labeled PMNs with PKH-26 labeled U937 cells for 1 h demonstrating an absence of yellow fluorescence (magnification x400). (C) Magnification of (A) showing yellow fluorescence in a PMN (arrow) and U937 cells (arrowheads) after 1 h co-culture (magnification x1000). (D) Co-culture of PKH-67-labeled PMNs and PKH-26-labeled U937 cells for 24 h; prominent yellow fluorescence is visible on PMNs (arrows) and U937 cells (arrowheads) (magnification x1000). (E) Results of FACS analysis demonstrating transfer of MHC class-II from human monocytes/macrophages to PMNs after co-culture for 2 h. (F) Immunoblot analysis of CD11a expression in U937 cell lysates stably transfected with specific CD11a siRNA or empty vector. (G) Densitometric quantification of CD11a in U937 cell lysates with/without siRNA knockdown by Western blot. (H) Total membrane transfer (expressed as mean fluorescence intensity, MFI) from U937 cells, with or without siRNA knockdown of CD11a, to PMNs after 2 h co-culture.

In addition to the trogocytosis observed between PMN and CD4^+^ T cells, yellow fluorescence indicating transfer of fluorescent markers, was also observed on the surface of both PMN and U937 cells after 1 h cell-cell contact between them (Fig 2A), but not when they were co-cultured in transwell chambers (Fig 2B). Yellow fluorescence on PMNs (arrows) and U937 cells (arrowheads) was more pronounced after overnight co-culture (Fig 2D) than after co-culture for 1 h (Fig 2C). More specifically, we found that HLA-II molecules were transferred from FITC-anti-HLA-II stained monocytes to PMNs (Fig 2E).

Masuda et al. [28] identified two mechanisms of trogocytosis: adhesion molecule-mediated and Fcγ receptor-mediated. Because integrin adhesion molecules, including CD11a/CD18, CD11b/CD18 and CD11c/CD18, important for immunological synapse formation are expressed on both PMNs and MNCs, we next determined the effects of integrins on trogocytosis between PMN and U937 cells. Since U937 cells express negligible CD11b, we used CD11a-specific siRNA to achieve efficient selective knockdown of CD11a in U937 cells (Fig 2F and 2G). Counterintuitively, siRNA knockdown of CD11a in U937 cells increased total membrane transfer from U937 to PMN cells compared to controls (Fig 2H). We suspect an unknown dominant compensatory mechanism operated between PMNs and U937 when the CD11a adhesion molecule was diminished on U937 cells; however, the mechanism behind this paradoxical increase in trogocytosis requires further investigation.

### Enhanced phagocytic activity and IL-8 production of PMN cells after cell-cell contact with T cells requires intact cytoskeletal structure, clathrin and Fcγ receptor for activation of the p38- and P44/42-Akt-MAP kinase signaling pathways

**Fig 3:**
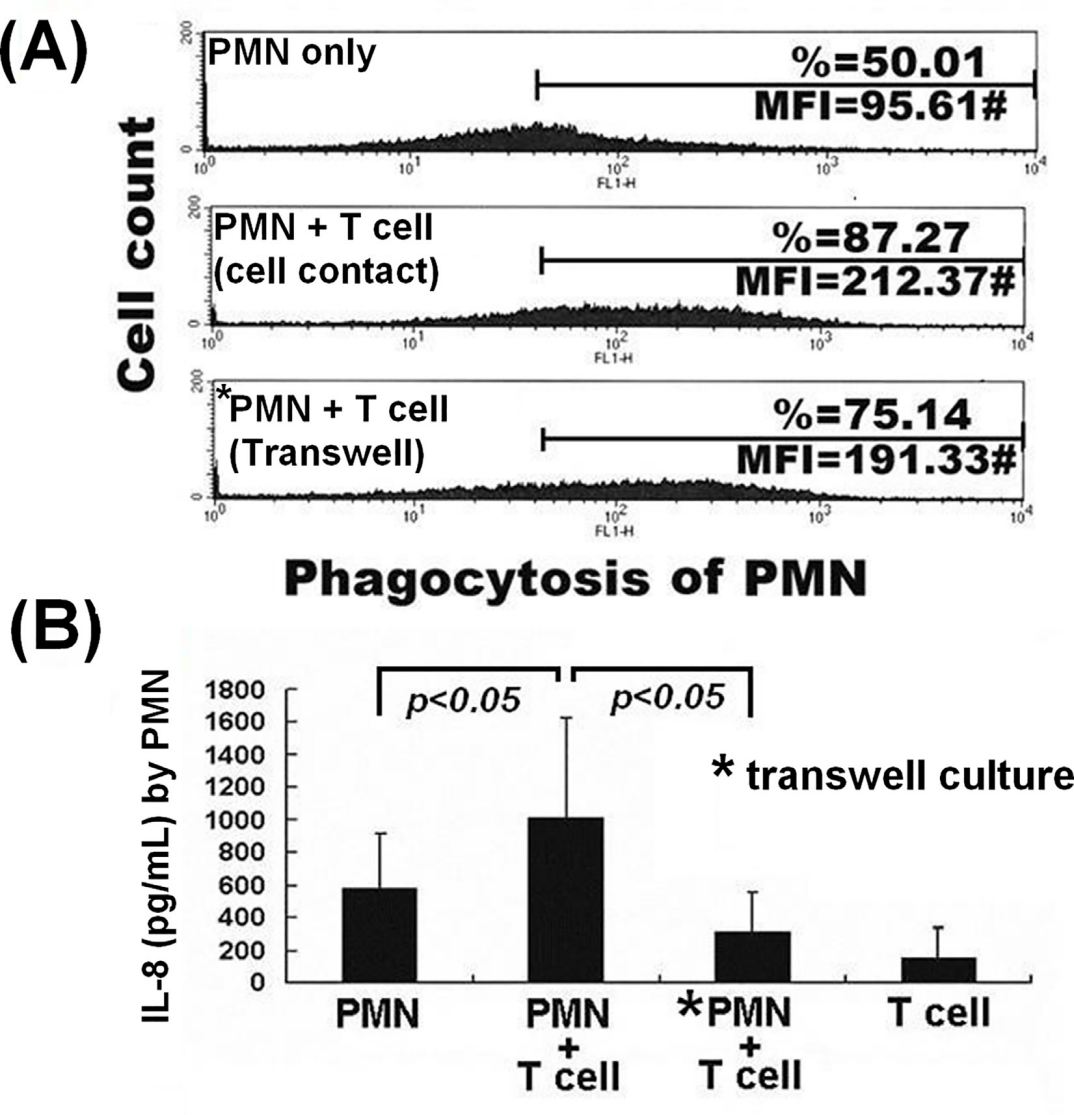
Enhanced phagocytic activity and IL-8 production of PMNs after cell-cell contact with autologous T cells. (A) A representative case from three independent experiments showing PMN phagocytotic activity (%) detected by Flow cytometry. (B) IL-8 concentration (pg/ml) in 6 h co-culture supernatants detected by ELISA (N = 8). *Indicates transwell co-culture.

To ascertain whether T cells can affect PMN functions after cell-cell contact, the two cell types were co-cultured either in conical tubes or transwell chambers. Mixing of PMN with T cells enhanced PMN phagocytosis (Fig 3A). Co-culture of PMN with T cells in conical tubes significantly enhanced IL-8 production, compared to that of PMNs either alone, or in transwell co-culture (*P <* 0.05, N = 8; Fig 3B). Collectively, these results show that T cells exert a contact-dependent enhancing effect on PMN function; however, trogocytosis is not the only activity dependent on cell-cell contact and many other mechanisms may occur as a result. Accordingly, we pre-incubated PMN with different membrane-active agents as potential trogocytosis-inhibitors to investigate correlation with PMN-enhancing activity. As shown in Table 2, latrunculin B suppressed PMN phagocytosis significantly (*P* < 0.05) and suppressed membrane transfer from T cells to PMNs moderately, but had no effect on IL-8 production. By contrast, MβCD (a cholesterol-depleting caveolin inhibitor) had no effect on phagocytosis or IL-8 production of PMNs. Pitstop-2 (specific inhibitor of clathrin-mediated endocytosis) exerted remarkable suppression of both trogocytosis (*P* < 0.01) and IL-8 production (*P* < 0.05), but not phagocytosis in PMNs. Human IgG moderately suppressed both trogocytosis and IL-8 production by PMNs (*P* < 0.05), but also had no effect on phagocytosis. These results suggest that clathrin and FcγR play a major role in PMN-MNC trogocytosis, in parallel with IL-8 production, and that an intact cytoskeletal structure is important for trogocytosis and phagocytosis, but not IL-8 production in PMNs. We deduce that PMNs acting as afferent cells capturing membrane from T cells require intact cytoskeletons and surface Fcγ receptors, as well as clathrin activation, but not caveola formation.

**Table 2:** Effects of different membrane integrity inhibitors on total membrane transfer from PKH26-labeled T cells to PMNs and PMN functions after 1 h pre-incubation detected by flow cytometry

PMN pre-incubation treatment	Membrane Transfer from T cells to PMN (%)		PMN phagocytosis (%)	IL-8 production by PMN (pg/ml)
Medium only		100	88.37 ± 8.42	932.40 ± 107.35
Latrunculin B (1 μM)		67.15 ± 10.65	40.12 ± 7.51*	969.83 ± 111.09
MβCD (5 mM)		120.50 ± 15.31	85.25 ± 6.34	983.64 ± 132.07
Pitstop-2 (30 μM)		40.13 ± 6.74**	80.08 ± 8.32	678.09 ± 90.45*
Human IgG (10 mg/ml)		62.51 ± 14.31*	81.58 ± 10.26	655.74 ± 88.34*

Latrunculin B, actin microfilament assemble inhibitor; MβCD (methyl-β-cyclodextrin), cholesterol depletion agent with anti-caveolin inhibitor; Pitstop-2, a specific inhibitor of clathrin-mediated endocytosis; Human IgG, inhibitor of FcγR binding

* p < 0.05 compared to culture medium control

** p < 0.01 compared to culture medium control

### Co-culture of PMN with T cells or monocytes decreases PMN apoptosis by suppressing extrinsic cell death signals

**Fig 4:**
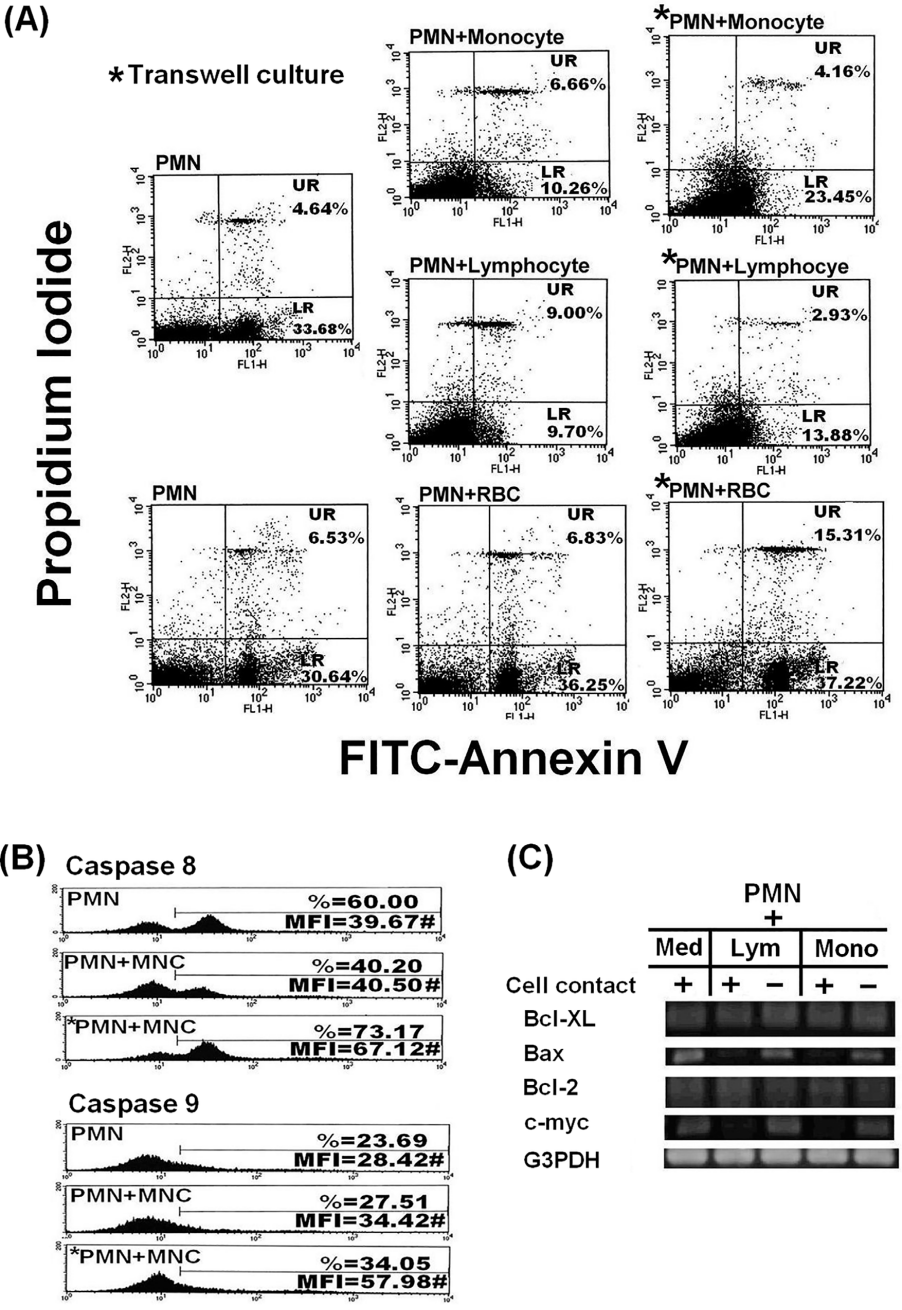
Co-culture of PMNs with lymphocytes or monocytes decreases PMN apoptosis via the anti-extrinsic pro-apoptotic pathway. (A) A representative case from four independent experiments showing the effect on PMN apoptosis after cell-cell contact with monocytes, lymphocytes or RBCs for 24 h. UR = late stage apoptosis. LR = early apoptosis. *Transwell chamber co-culture. (B) A representative case from three independent experiments to detect caspase 8 (above) and caspase 9 (below) by FLICA. In each case, the upper panel shows results from PMNs cultured alone, whereas the middle and bottom panels are results from PMNs and MNCs co-cultured or cultured in transwell chambers, respectively. (C) Detection of pro-apoptotic (*BAX* and *MYC*) and anti-apoptotic gene (*BCL2L1* and *BCL2*) mRNA expression in PMN by RT-PCR after cell-cell contact co-culture with lymphocytes or monocytes/macrophages for 2 h. Two independent experiments were conducted.

PMNs and MNCs may engage in cross-talk and mutually modulate their functions at sites of chronic infection, inflammation or in tumor tissues [29–34]. In addition, Poupor et al. [17] reported that spontaneous membrane transfer among certain homotypical leukemia cell lines transduces cell survival signals [17]. To further elucidate the molecular basis of the enhancement of PMN activities by trogocytosis with MNCs, we co-cultured PMNs with monocytes or T cells for 2 h and noted a significant reduction in PMN apoptosis compared to transwell co-culture or co-culture with RBCs (Fig 4A). FLICA assays produced double peaks only in PMNs stained with caspase 8. Decreased intracellular caspase 8, but not caspase 9 was evident after PMN-MNC cell contact (Fig 4B). After cell-cell contact with lymphocytes or monocytes, PMN expression of pro-apoptotic *BAX* and *MYC* was decreased, whereas that of anti-apoptotic *BCL2L1* and *BCL2* were unchanged (Fig 4C). These results indicate that PMN-MNC contact suppressed extrinsic apoptotic molecules, including *BAX*, *MYC* and caspase 8 to reduce PMN apoptosis via a contact-dependent mechanism.

### Signaling pathways activated in PMNs after co-culture with lymphocytes and monocytes

**Fig 5:**
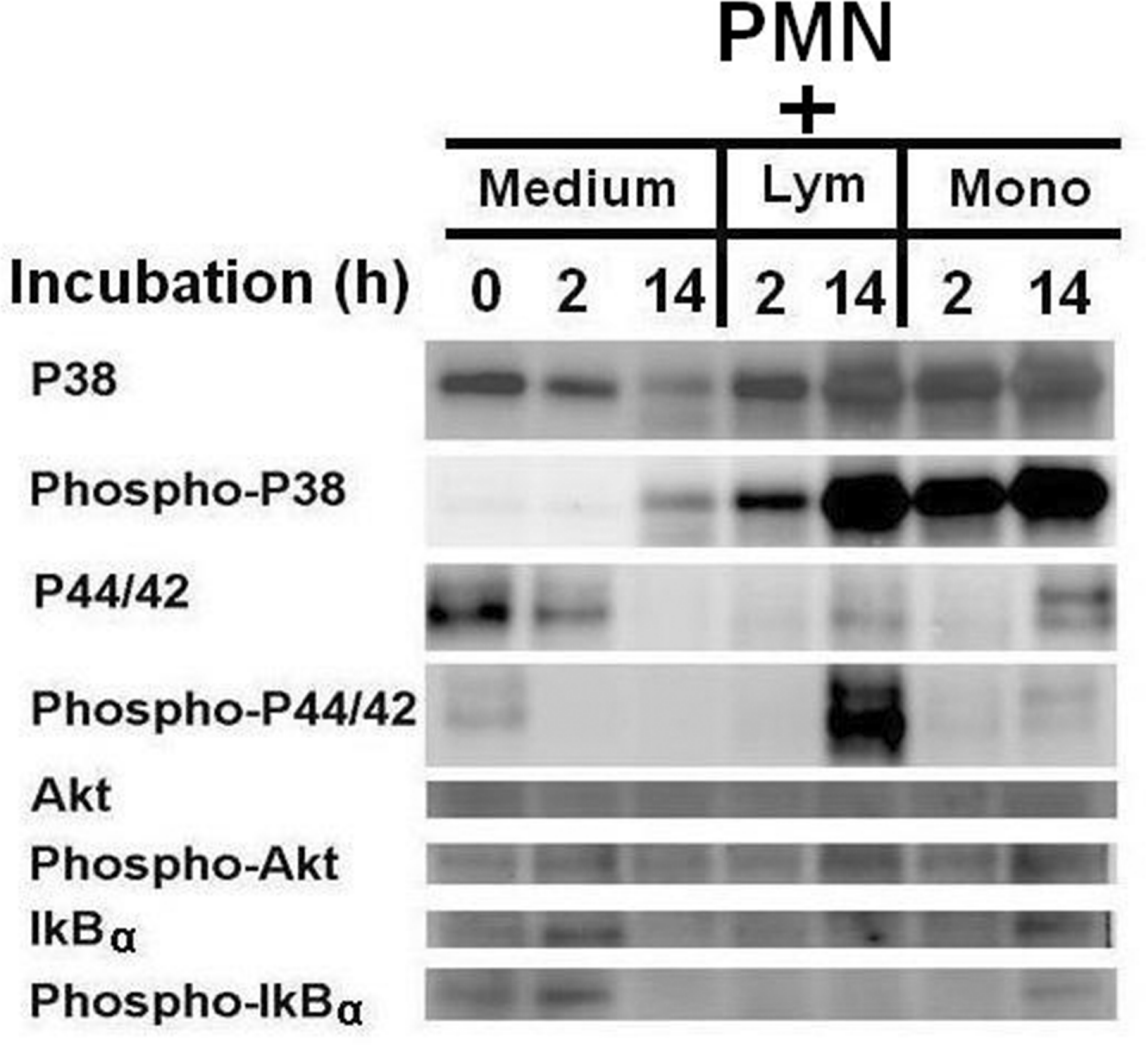
Detection of intracellular signaling molecule expression in PMN by Western blot after co-culture with lymphocytes (Lym) or monocytes (Mono) for 2 h and 14h.

We hypothesized that activation signals in PMNs would be elicited via trogocytosis with MNCs, resulting in the observed reduction in extrinsic apoptotic signals. We next measured phosphorylation of p38- and p44/42-Akt-MAPKs, and IκB molecules involved in cell activation and apoptosis in PMNs alone or when co-cultured with T cells or monocytes, at different time points (0, 2, 14 h). As shown in Fig 5, phosphorylation of p38, p44/42 and Akt was increased, whereas phosphorylation of IκB-α was decreased, in co-cultures of PMN with CD4^+^ T cells or monocytes. In conclusion, T cells and monocytes can enhance PMN functions by increasing p38- and p42/44-Akt MAPK phosphorylation. Decreased IkB phosphorylation may be a consequence of decreased PMN apoptosis.

### Molecules in the immunological synapse and the non-synaptic chemokine receptor CXCR1 are transferred from PMNs to autologous CD4^+^ T cells after trogocytosis

**Fig 6:**
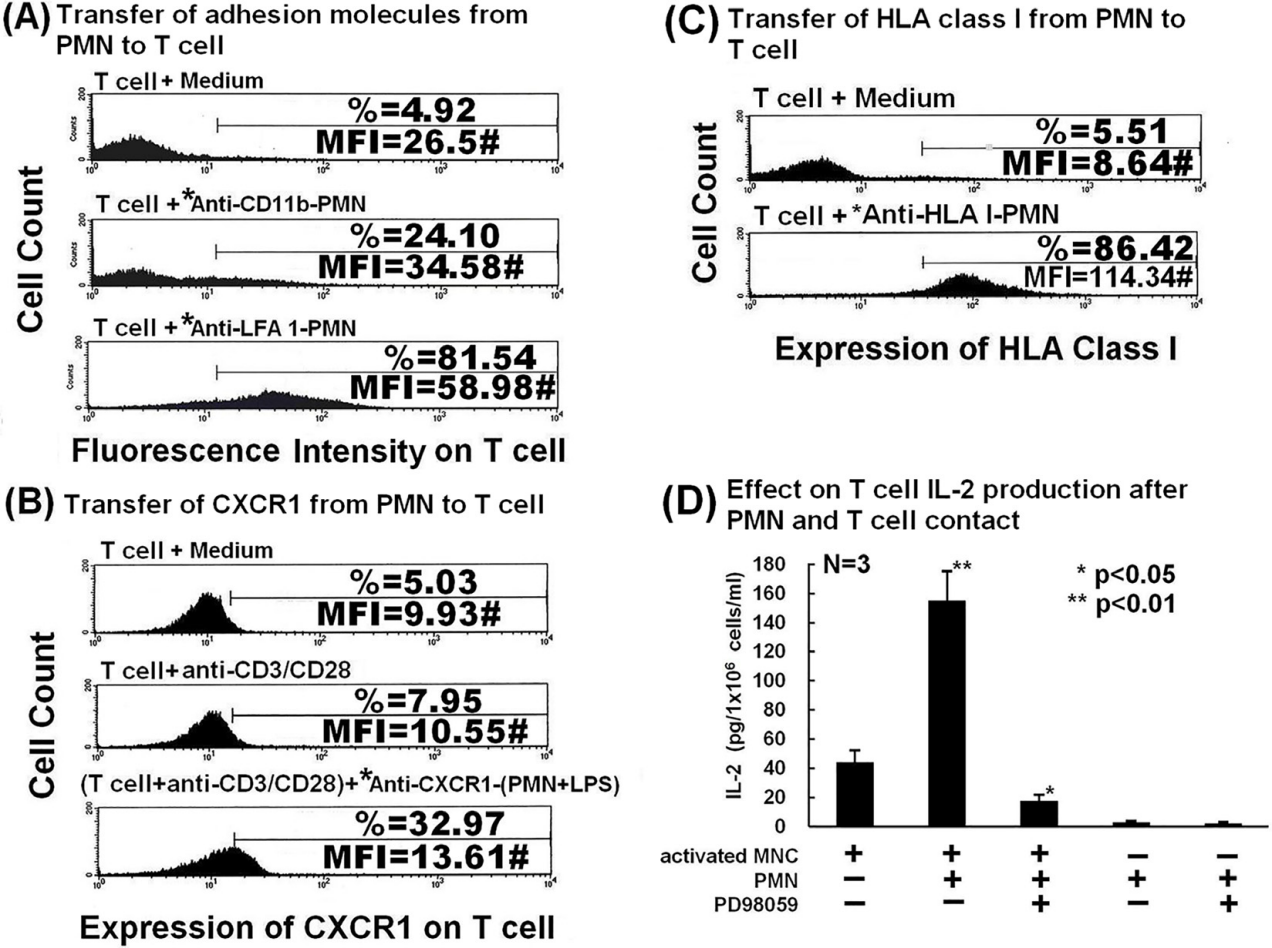
Detection of transfer of specific surface-expressed molecules from PMN to T cells and effects on IL-2 production by anti-CD3/anti-CD28 activated MNCs. Results of FACS analysis demonstrating (A) transfer of adhesion molecules CD11b and LFA-1, (B) transfer of CXCR1, and (C) transfer of HLA class-I from PMN to T cells after co-culture for 2 h. *FITC-antibody-stained PMNs. (D) Effects of autologous PMNs and MAP kinase inhibitor PD98059 on IL-2 production by activated human MNCs.

PMNs, do not only act as afferent cells receiving membrane from other immune cells, they can also act as efferent cells, transferring membrane molecules by cell-cell contact. To identify specific surface-expressed molecules transferred from PMN to CD4^+^ T cells, we first focused on molecules in the immunological synapse, including MHC antigens and adhesion molecules. Fig 6 shows that adhesion molecules CD11b and LFA-1 (Fig 6A), CXCR1 (Fig 6B) and HLA-I (Fig 6C) were transferred from PMNs to CD4 ^+^T cells. Thus, molecules in both central and peripheral supramolecular activation clusters of immunological synapses on PMN, as well as CXCR1, can be transferred from PMN to CD4 ^+^T cells after cell-cell contact. Together with the transfer of HLA-II molecules from monocytes to PMNs (Fig 2E), we conclude that specific molecules both within and outside (CD4, CD16 and CXCR1) the immunological synapse are exchanged between PMNs and MNCs. The specific surface molecules transferred among PMNs, T cells and monocytes are summarized in Table 3.

**Table 3:** Specific surface molecule transfer among PMN, T cells and monocytes.

Donor cell	Recipient cell	Surface molecule	Detection Method
PMN	T cell	HLA-I	Flow cytometry
PMN	T cell	CD11b	Flow cytometry
PMN	T cell	CXCR1	Flow cytometry
PMN	T cell	LFA-1	Flow cytometry
T cell	PMN	HLA-I	Flow cytometry
Monocyte	PMN	HLA-II	Flow cytometry
T cell	PMN	CD4	Confocal microscopy
PMN	T cell	CD16	Confocal microscopy

### Total membrane transfer from PMNs to MNCs augments IL-2 production by activated MNCs and this can be suppressed using Rottlerin and PD98059

To further understand the immunological effects of membrane transfer from PMNs to MNCs, IL-2 production by anti-CD3/anti-CD28 activated MNCs was assessed in the presence or absence of trogocytosis with PMNs. Fluorescence captured by MNCs from PKH-67-labeled PMNs was compared after pre-incubation of PMNs with or without inhibitors. Rottlerin (a PKC and CaM kinase III inhibitor) and PD98059 (a MAPK2-specific inhibitor) significantly suppressed PMN-MNC trogocytosis, consistent with data in Fig 5, showing that p38- and p44/42-Akt-MAPKs signals were involved in PMN-MNC trogocytosis. Accordingly, we measured the effects of PD98059 on IL-2 cytokine production by anti-CD3/anti-CD28-stimulated MNCs in the presence/absence of autologous PMNs. As shown in Fig 6D, activated MNCs produced more IL-2 after mixing with PMNs compared to culture alone (154.9 ± 20.4 pg/ml vs. 43.7 ± 8.6 pg/ml). This enhancement was remarkably suppressed by PD98059 (19.2 ± 6.6 pg/ml). We next sought to investigate the activation signals involved in membrane transfer from PMNs to MNCs. Table 4 shows the pharmacological effects and proportional inhibition of different signaling pathway inhibitors on membrane transfer from PMNs to MNCs. In conclusion, MAP kinase and PKC signaling pathways that are transduced after PMN-MNC trogocytosis can affect the functions of both PMNs and MNCs.

**Table 4:** Effects of different inhibitors on PMN-MNC trogocytosis detected by flow cytometry.

Drug added (final concentration)	Action	Membrane transfer from PKH67-PMN to MNC (%)
None	−	100.00
Rottlerin (100 μM)	PKC and CaM kinase III inhibition	40.30 ± 12.91*
PP2 (10 μM)	Src kinase inhibition	89.61 ± 16.85
NLA (100 μM)	NO synthase inhibition	106.2 ± 5.96
EGTA (100 μM)	Calcium chelating agent	90.74 ± 13.26
Thapsigargin (100 nM)	Intracellular calcium release	116.10 ± 17.94
Wortmannin (100 nM)	PI3K inhibition	129.65 ± 7.87
PD98059 (100 μM)	MAPKK inhibition	36.53 ± 6.65*

NLA, N-nitro-L-arginine; EGTA, *ethylene glycol tetra-acetic acid*; NO, nitric oxide; PI3K, phosphoinositide 3-kinase; MAPKK, *mitogen-activated protein kinase kinase*; PKC, protein kinase C; CaM, calmodulin; PMN, polymorphonuclear neutrophils; MNC, mononuclear cells.

*denotes *p*<0.05

### Decreased total membrane transfer from PMNs to MNCs suppressed IL-2 production by activated MNCs in patients with active SLE

**Fig 7:**
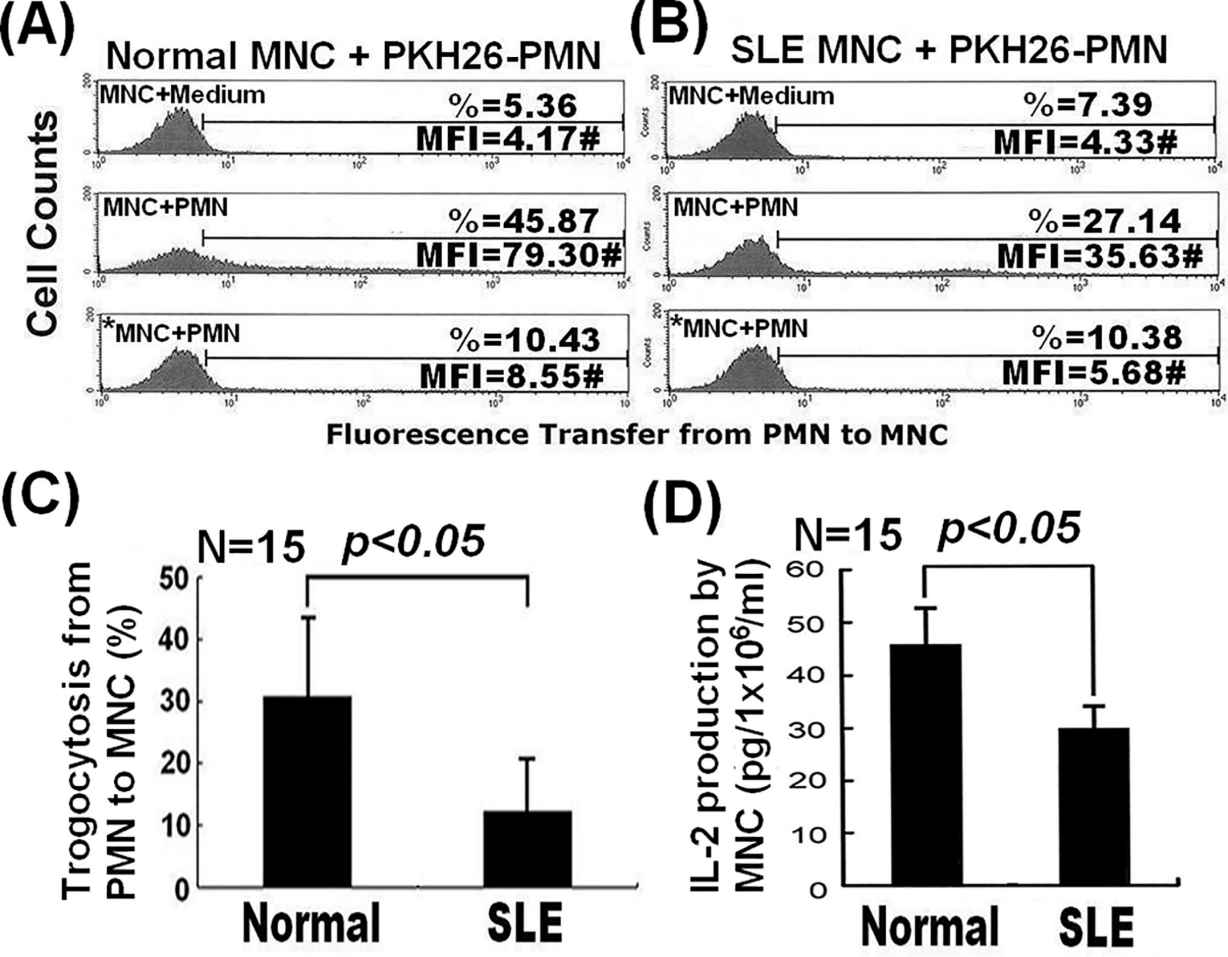
Comparative total membrane transfer from PMNs to MNCs and effects on IL-2 production by MNCs in active SLE versus healthy controls. Results of FACS analysis of representative cases showing (A) the proportion of total membrane transfer from PMNs to MNCs using cells from a healthy individual and (B) the proportion of total membrane transfer from PMNs to MNCs in a patient with active SLE. (C) Comparative proportion of total membrane transfer from PMNs to MNCs in patients with active SLE versus healthy controls. (D) Comparative IL-2 production in normal and active SLE groups.

It is hypothesized that the Th1 pathway is suppressed in patients with active SLE by an overactive Th2 pathway. Therefore, we measured the membrane transfer from PMNs to MNCs, together with IL-2 production by activated MNCs, in samples from patients with active SLE. Representative examples of healthy and SLE PMN-MNC trogocytosis are shown in Fig 7A and 7B, respectively. We found that total membrane transfer from PMNs to MNCs in samples from patients with active SLE was significantly lower than in those from healthy controls (Fig 7C). Functionally, SLE autologous anti-CD3/anti-CD28-activated MNCs produced less IL-2 than those from the healthy group (Fig 7D).

## Discussion

Intercellular communications among immune cells involve many mechanisms, including formation of immunological synapses, tunneling nanotubes, trogocytosis, and release of exosomes [23,34]. Trogocytosis has been described among different immune cells, including CD4^+^ T cells, CD8^+^ T cells, γδT cells, B cells, NK cells, dendritic cells, and monocytes/macrophages, and may serve to increase plasticity in the immune system [14,16,19,35–40]. However, the nature and biological roles of trogocytosis are still unclear [17, 19]. We previously demonstrated that surface-expressed lactoferrins are transferred from PMNs to CD4^+^ T cells in a contact-dependent manner to modulate Th1/Th2 cytokine production by activated MNCs in healthy individuals and patients with SLE [20]. It is thought that PMNs and T lymphocytes may interact directly to transmit signals, in addition to communication through cytokine production [36, 39–40]. This study revealed several new insights: (1) Fig 1 and Fig 2 clearly demonstrate trogocytosis between PMNs and CD4^+^ T cells and between PMNs and monocytes/macrophages (U937 cells); (2) Molecules in central (HLA class-I and -II) and peripheral (adhesion molecules CD11b and LFA-1) supermolecular clusters of the immunological synapse and non-synaptic molecules (CXCR1 and CD16 on PMN, and CD4 on CD4^+^ T cells) can be exchanged between PMNs and MNCs by cell-cell contact; (3) Membrane transfer from MNCs to PMNs suppresses extrinsic apoptotic pathway molecules, including *BAX*, *MYC* and caspase 8 in PMNs; (4) Membrane transfer from MNCs to PMNs transduces activation signals to enhance PMN phagocytosis and IL-8 production via the p38- and p44/42-Akt MAPK signaling pathways; and (5) Decreased trogocytosis from PMNs to MNCs resulted in suppression of IL-2 cytokine production by anti-CD3/anti-CD28 activated MNCs in patients with active SLE, relative to healthy control subjects. We did not measure trogocytosis between B cells and other innate or adaptive immune cells in the present study, because the number of B cells in peripheral blood is too low to conduct these experiments. We intend to conduct trogocytosis experiments using B cell leukemia or lymphoma cell lines in the near future.

Another novel finding in the present study is that membrane capture by PMNs (afferent limb of trogocytosis) from T cells required intact cytoskeletons, clathrin activation and Fcγ receptors (Table 2). These molecules are important for signal transduction and many plasma membrane activities. In contrast, membrane release (efferent limb of trogocytosis) from PMNs to MNCs depended on MAP kinase and PKC signaling pathways (Table 4). To the best of our knowledge, no similar results have previously been reported. Clearly, additional investigations will be required to confirm these findings.

Our results are similar to those of another study showing decreased expression of HLA-G, a non-classical class I HLA molecule with an important role in regulating the immune response, on macrophages/mature dendritic cells and diminished trogocytosis to autologous lymphocytes in patients with SLE [41]. Many studies have demonstrated that PMNs and lymphocytes coexist and can interact at sites of chronic inflammation, infection, or allergic reactions *in vivo* [13, 29–32]. Some investigators suggest that dendritic cells may up-regulate MHC class-II and CD69 expression on PMNs after cell contact and induce exhibition of dendritic cell-like phenotypes in PMNs [29, 38]. These observations are congruent with our findings that MHC-II can be transferred from monocytes to PMNs after cell-cell contact (Fig 2E). Accordingly, PMNs and MNCs may engage in bi-directional cross-talk through trogocytosis to modulate cell functions [37], as an alternative to cytokine production [1, 2] and exosome release [23]. We also confirmed that trogocytosis between PMNs and MNCs is a specific process, because no membrane transfer occurred between RBCs and PMNs or MNCs, as described in our previous report [22].

The immunological synapse is an important site for trogocytosis after PMN and MNC contact, as CD31, CD49b, CD80, CD86, ICAM-1, CD61, and MHC class-I and -II are exchanged by trogocytosis among immune cells [14,15, 34, 37]. We observed that molecules in the central (HLA-I and HLA-II) and peripheral (CD11b and LFA-1) supramolecular clusters were exchanged from PMNs to T cells after close contact (Fig 6A–6C). Interestingly, CD11a gene knockdown by siRNA in U937 cells enhanced total membrane transfer from U937 cells to PMNs (Fig 2H). Although the mechanism underlying this paradoxical enhancement is not clear, we speculate that an unknown dominant compensatory feedback mechanism may operate to enhance total PMN-macrophage trogocytosis; investigations are underway to ascertain the mechanism. Nonetheless, these data indicate the importance of the immunological synapse in immunocyte trogocytosis. Significant suppression of membrane transfer from PMNs to MNCs by Rottlerin or PD98059 is further support for the involvement of PKC/calmodulin-dependent protein kinase and MAPK pathways in trogocytosis (Table 4). Decreased total membrane transfer from PMNs to MNCs in patients with SLE suppressed activated mononuclear IL-2 cytokine production (Fig 7D). The cause of decreased trogocytosis from PMN to MNC in active SLE remains unclear; however, recent reports demonstrate that epratuzumab (anti-CD22) and a bi-specific hexavalent monoclonal antibody comprising epratuzumab and veltuzumab (a humanized anti-CD20 monoclonal antibody), exhibit enhanced Fc/FcR-dependent trogocytosis from B cells to different immune cells, resulting in a remarkable reduction in B cell surface expression of CD19, CD20, CD21, CD22, and CD79b, as well as the key cell adhesion molecules CD44, CD62L, and β7-integrin [42, 43]. This may lead to a substantial reduction in adhesion molecule-mediated trogocytosis. In addition, anti-neutrophil antibodies such as anti-SSB/La antibodies in the sera of patients with active SLE may further deplete membrane molecules transferrable from PMNs to MNCs by impairing PMN functions *in vivo* [44]. This may lead to decreased total membrane transfer from PMNs to MNCs and cytokine production after cell-cell contact in patients with active SLE.

MAP kinases comprise three families, p38, JNK1–3, and p44/42, which transduce activation signals from cell membranes to the nucleus in response to external stimuli [45]. Phosphorylation of the p44/42-Akt-MAPK pathway renders cells resistant to apoptosis. In contrast, phosphorylation of the p38-Akt-MAPK pathway results in apoptosis or survival, depending on the cell types and milieu [46]; in PMNs, p38 phosphorylation may delay cell apoptosis [47,48]. We have shown phosphorylation of p38- and p44/42-MAPKs to be prominent in PMNs after overnight co-culture with monocytes or lymphocytes (Fig 5). Increased Akt phosphorylation may produce survival signals that inhibit cell apoptosis induced by TNF-α [49]. Thus, MNCs transduce both survival and activation signals to PMNs via trogocytosis to suppress apoptosis and enhance cell functions.

The significant anti-apoptotic effects observed after 24 h co-culture of PMNs and MNCs (Fig 4C) are compatible with the neutrophilia and delayed neutrophil apoptosis observed in chronic inflammatory conditions and autoimmune disease [31–33]. We discovered that MNCs delayed PMN apoptosis via decreased expression of extrinsic death molecules, including *BAX*, *MYC*, and caspase 8 (Fig 4B and 4C). These interesting findings are consistent with the observation that increased membrane transfer between homotypical lymphoma cell lines increases cell survival [17].

The true biological significance of trogocytosis remains controversial. One theory argues that trogocytosis among immune cells enhances immune plasticity by sustaining autonomous activation, even in the absence of interactions with other cells [49], although MAPKs are activated by many factors such as mitogens, growth factors, oxidative stress, osmotic stress, and ultraviolet and gamma-ray radiation [50]. Increasing evidence for interactions between PMNs and T cells *in vivo* has been found in different physiological and pathological conditions. PMNs with CCR7 expression enter into lymphoid organs, attracted by the chemokines CCL19 and CCL21, released from lymph nodes to modulate the adaptive immune response [51]. Furthermore, evidence for co-localization and interactions of PMNs with T cells has been reported at sites of persistent bacterial infection, chronic inflammation, or in tumors [40]. In the present study we identified direct cell-cell contact as another stimulus that activates MAPK signaling. Reduced membrane transfer from PMNs to MNCs resulted in suppressed autologous mononuclear IL-2 cytokine production in patients with active SLE as well as in the presence of PD98059. The molecular basis of decreased trogocytosis between PMNs and MNCs in active SLE clearly warrants further investigation.

In conclusion, PMNs and MNCs exchange various molecules in both immunological synapse and non-synaptic sites during trogocytosis. We confirmed that MNCs transduce survival and activation signals to PMNs and enhance PMN functions. In addition, PMNs can enhance mononuclear cell IL-2 cytokine production via MAP kinase and PKC signaling pathways. Defective membrane transfer from PMNs to MNCs in patients with active SLE suppresses mononuclear IL-2 cytokine production by autologous PMNs.

## Supporting Information

S1 Dataset Individual data points in this study. (XLSX)
